# Cold-Induced Changes in the Protein Ubiquitin

**DOI:** 10.1371/journal.pone.0037270

**Published:** 2012-06-21

**Authors:** Min-Kyu Cho, ShengQi Xiang, Hai-Young Kim, Stefan Becker, Markus Zweckstetter

**Affiliations:** Department for NMR-Based Structural Biology, Max-Planck-Institute for Biophysical Chemistry, Göttingen, Germany; Spanish National Cancer Center, Spain

## Abstract

Conformational changes are essential for protein-protein and protein-ligand recognition. Here we probed changes in the structure of the protein ubiquitin at low temperatures in supercooled water using NMR spectroscopy. We demonstrate that ubiquitin is well folded down to 263 K, although slight rearrangements in the hydrophobic core occur. However, amide proton chemical shifts show non-linear temperature dependence in supercooled solution and backbone hydrogen bonds become weaker in the region that is most prone to cold-denaturation. Our data suggest that the weakening of the hydrogen bonds in the β-sheet of ubiquitin might be one of the first events that occur during cold-denaturation of ubiquitin. Interestingly, the same region is strongly involved in ubiquitin-protein complexes suggesting that this part of ubiquitin more easily adjusts to conformational changes required for complex formation.

## Introduction

Conformational changes are essential for protein-protein and protein-ligand recognition [1]. For efficient interaction, it is assumed that a certain amount of plasticity in the active site is required to accommodate its ligand in combination with a conformational change induced by the ligand at the binding site [2]. In addition, intrinsic dynamics can play a key role such that the ligand will bind selectively to the active conformation, thereby biasing the equilibrium toward the binding conformation [3]. NMR dipolar couplings observed in the protein ubiquitin in combination with 3D structures of ubiquitin in complex with binding partners have recently provided support for the presence of binding-relevant conformations in the native state [4].

The stability of protein conformations can be probed by exposing the protein to external perturbations such as high pressure, acidic pH, chemical denaturants or high and low temperature. Particularly interesting is cold denaturation of proteins. When the temperature of the solution is reduced sufficiently without freezing, proteins can be cold denatured without the need for chemicals that would potentially interfere with the ensemble of conformations present in solution. Thus, insight into the origin of the cooperativity of protein folding and the nature of partially folded states might be obtained [5], [6], [7], [8], [9], [10]. The predicted cold-denaturation temperature of proteins is typically 20 K or more below the equilibrium freezing point of water [9], [11].

Measurements in supercooled water – that is at temperatures well below the freezing point of water but above those at which proteins cold-denature - can provide a wealth of information about protein structure, dynamics and hydration [5], [12]. In particular, interconversion between different conformations that causes averaging of spectroscopic probes at higher temperatures is slowed down. This can be used to reduce the flip-broadening of aromatic NMR lines [13], [14]. In addition, the temperature-dependent exchange of protons in RNA duplexes is reduced in supercooled water, allowing the observation of non-base-paired imino protons of RNA [15]. We previously showed that supercooled water can be used to slow down interconversion between different conformations of the protein α-synuclein in its monomeric, disordered state thereby allowing access to its intrinsic residual secondary structure [16].

In the current study, we investigated the structure of the 76-residue protein ubiquitin at low temperatures in supercooled water using NMR spectroscopy. We compare our findings of cold-induced changes with regions that are dynamic and involved in protein interactions in the native state of ubiquitin.

**Figure 1:**
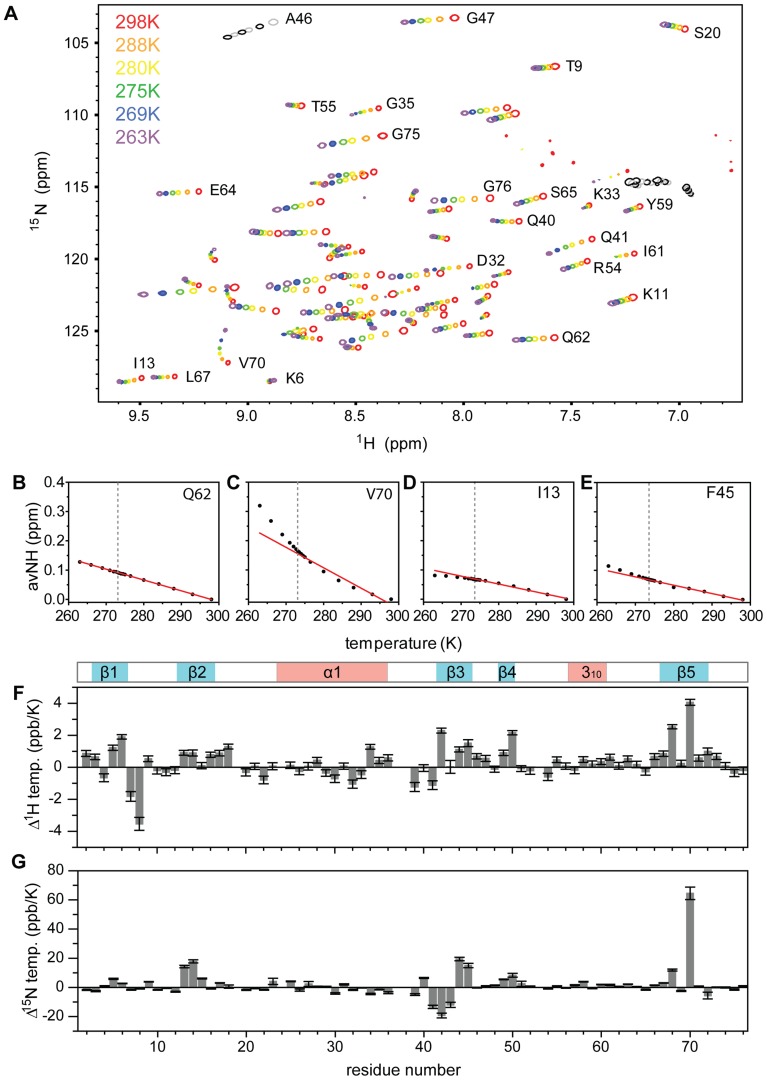
Non-linearity of the [^1^H,^15^N] chemical shift changes in ubiquitin at decreasing temperatures. (A) 2D [^1^H,^15^N]-HSQCs of ubiquitin for temperatures from 298 K to 263 K. Selected resonance assignments are indicated. (B–E) Weighted average [^1^H,^15^N] chemical shift changes as a function of temperature for selected residues. The red line shows the straight line fit to the data in the range 298K-273K. Differences between amide proton (F) and nitrogen (G) temperature coefficients in the range 273 K-263 K and 298 K-273 K. The location of helices and β-strands is schematically shown above.

**Figure 2:**
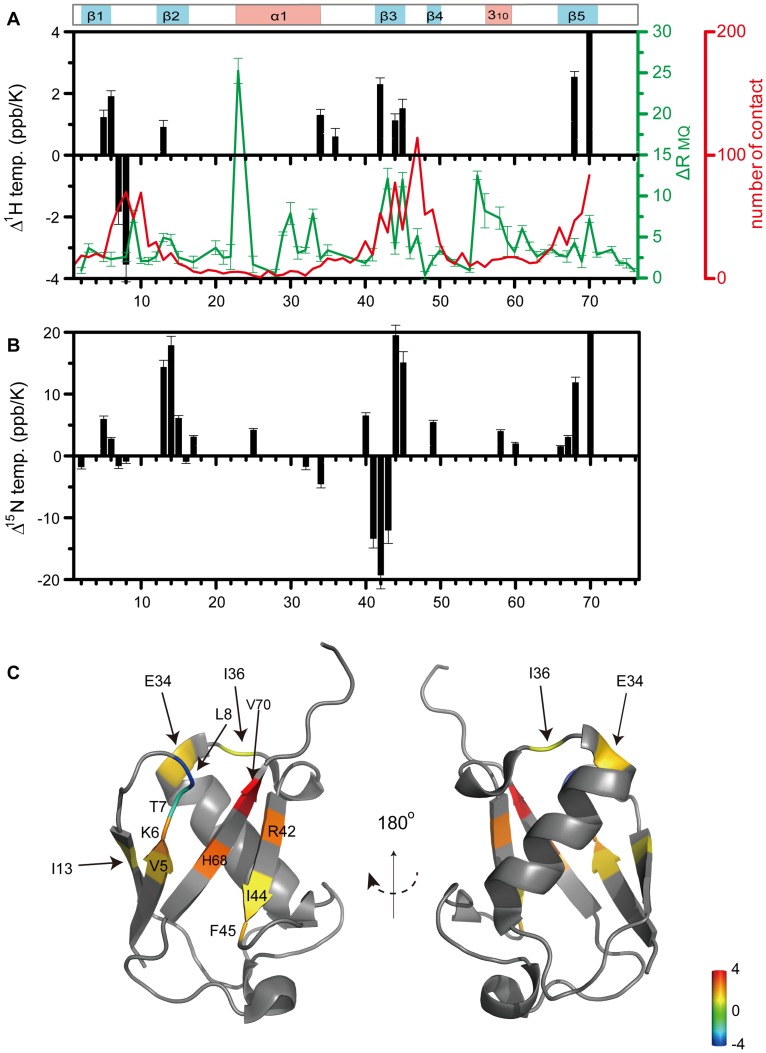
Cold-induced changes in the amide proton temperature coefficients. (A, B) Residue-specific differences in the amide proton (A) and amide (B) temperature coefficients above (298 K-273 K) and below 273 K (273 K-263 K) (black bars). Only values for those residues are shown for which p-value <0.02% in the F-test. For comparison, the number of ubiquitin-binding protein contacts per residue (red) and the amide ^1^H-^15^N relaxation rate constants for multiple-quantum coherences, ΔR_MQ_, measured for ubiquitin at 280 K (green) are shown [28]. The location of helices and β-strands is schematically shown above. (C) 3D structure of ubiquitin highlighting residues that showed statistically significant differences in the amide proton temperature coefficients above and below 273 K (as shown in A). The color coding follows the magnitude of Δ^1^H(temp) shown in A).

**Figure 3:**
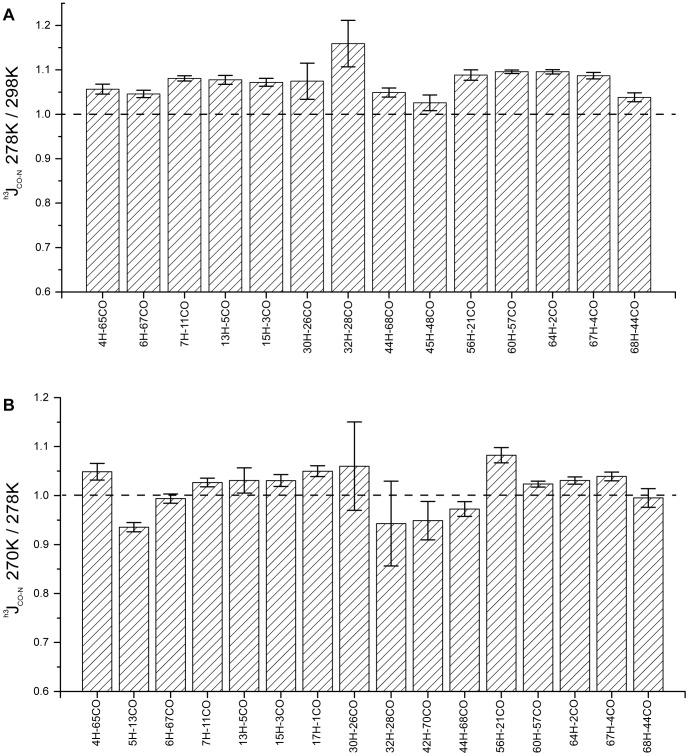
Weakening of hydrogen bonds in supercooled solution. Ratios of ^h3^J_NC’_ trans-hydrogen bond scalar couplings at 278 K and 298 K (A), and at 270 K and 278 K (B). Only residues not affected by signal overlap were included. Errors were calculated on the basis of the signal-to-noise ratio of the cross and reference peak. On the x-axis the donor and acceptor residue are indicated.

## Results and Discussion

Figure 1A shows two-dimensional [^1^H,^15^N]-heteronuclear single quantum coherence (HSQC) spectra of ubiquitin during a stepwise decrease in temperature from 298 K to 263 K. Many residues such as Q62 displayed linearly changing chemical shifts across the whole temperature range (Figure 1B–E and Figure S1), as expected for stably folded structures and in agreement with the known hydrogen bonds of ubiquitin [17], [18]. In contrast, for several other residues the chemical shift changes deviated from a linear temperature dependence (Figure S1). R42, I44, H68 and V70 showed deviations from linearity already in the range from 298 K to 273 K. For several other residues, however, chemical shift changes could be well approximated by a linear function above and below 273 K, but experienced strong non-linearity close to 273 K (Figure 1B–E and Figure S1). We therefore determined two temperature coefficients, one in the range from 298 K to 273 K and the other from 273 K to 263 K (Figures 1F,1G and S2; Table S1). Note that the linear fit is only an approximation to approximately quantify the strength of the temperature-induced changes. Statistical analysis of the changes in the amide protein chemical shifts using F-tests with a p-value of <0.02% identified in total 12 residues: 5–8, 13, 34, 36, 42, 44, 45, 68 and 70 (Figures 1F, 2A). The same analysis for nitrogen chemical shifts highlighted similar regions, but revealed non-linearity for a few more residues and highlighted the changes in the regions of residues 13–14, 42–45 and 68–70 (Figures 1G, 2B). Mapping of the changes in amide proton temperature coefficients onto the 3D structure of ubiquitin showed that the β-sheet of ubiquitin is most strongly affected (Figure 2C). In particular, residues connected by hydrogen bonds across the β-sheet (I13-V5/K6-H68-I44) appear as a continuous ridge.

**Figure 4:**
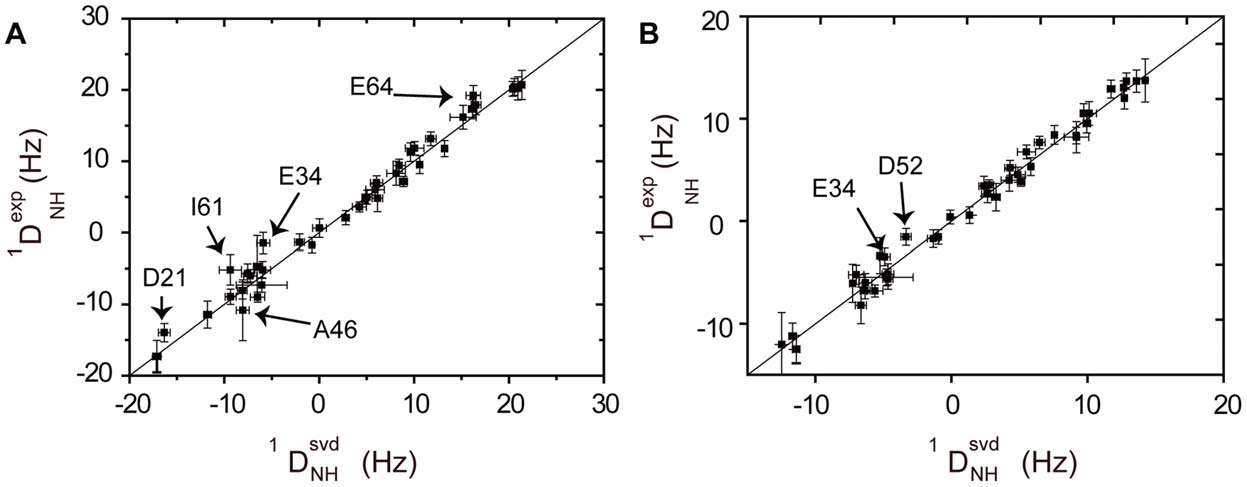
Ubiquitin remains folded in supercooled solution down to 263 K. (A, B) Correlation between experimental backbone [^1^H,^15^N] residual dipolar couplings observed at (A) 270 K and (B) at 278 K with values calculated by singular-value decomposition from the solution NMR structure of ubiquitin (PDB entry 1D3Z). Residues deviating from a linear fit are marked.

Non-linear chemical shift changes reflect the temperature dependence of the populations of states of different free energies and these states can reflect any number of physical changes, including hydrogen bonding, changes in electrostatics, changes in dihedral angles and packing [19]. Moreover, amide proton temperature coefficients depend on the strength of hydrogen bonds, as well as other factors such as deshielding due to conformational changes in nearby aromatic groups [17]. We therefore directly probed the sensitivity of the hydrogen bonds of ubiquitin to low temperature using three-bond trans-hydrogen bond scalar couplings [20], [21], [22]. To obtain sufficient accuracy even at low temperatures where the rotational diffusion is reduced, the measurements were performed at a concentration of 5 mM of ^2^H/^15^N/^13^C-labeled ubiquitin. In addition, only cross-peaks not affected by signal overlap were included into the analysis. Upon lowering the temperature from 298 K to 278 K the magnitude of the ^h3^J(CO-N) trans-hydrogen bond scalar coupling increased in a rather uniform manner by about 4–8% (Figure 3A). This is in line with previous observations that reported an increase in ^h3^J(CO-N) values with decreasing temperatures [18]. When the temperature was decreased to 270 K the ^h3^J(CO-N) values of the amide protons of residues 4, 7, 15, 17, 56, 60, 64 and 67 further increased (Figure 3B), while for residues 13, 30 and 32 the errors were too large to reliably identify changes (Figure 3B). In contrast, for the amide protons of residues 5, 6, 42, 44 and 68 the ^h3^J(CO-N) values decreased or were unchanged within the experimental error. The decreased/unchanged magnitude of ^h3^J(CO-N) for these amide protons demonstrates that the corresponding hydrogen bonds become weaker in supercooled solution, in line with increased amide proton temperature coefficients (Figure 1F). The amide proton of Thr7, which is hydrogen bonded to the carbonyl of residue 11 in the native state of ubiquitin, did not show a decrease in the magnitude of ^h3^J(CO-N) (Figure 3B). Moreover, the amide proton temperature coefficient of Thr7 became more negative when the temperature was reduced below 273 K (Figure S2), suggesting that the non-linear chemical shift changes of Thr7 are not related to changes in the strength of the hydrogen bond. Note that although Thr7 is involved in a hydrogen bond its amide proton temperature coefficient is approximately −5 ppb/K for temperatures above 273 K and thus below the value of −4.6 ppb/K that is usually used as cutoff for the identification of hydrogen bonds [17]. Taken together our data demonstrate that at low temperatures in supercooled solutions hydrogen bonds in the β-sheet of ubiquitin are weakened. At the same time it should be kept in mind that the lengthening (weakening) of a hydrogen bond does not necessarily lead directly to a loss of stability of the protein. Compensating enthalpic and entropic effects, such as better repacking of the core can mitigate changes in hydrogen bonding.

**Figure 5:**
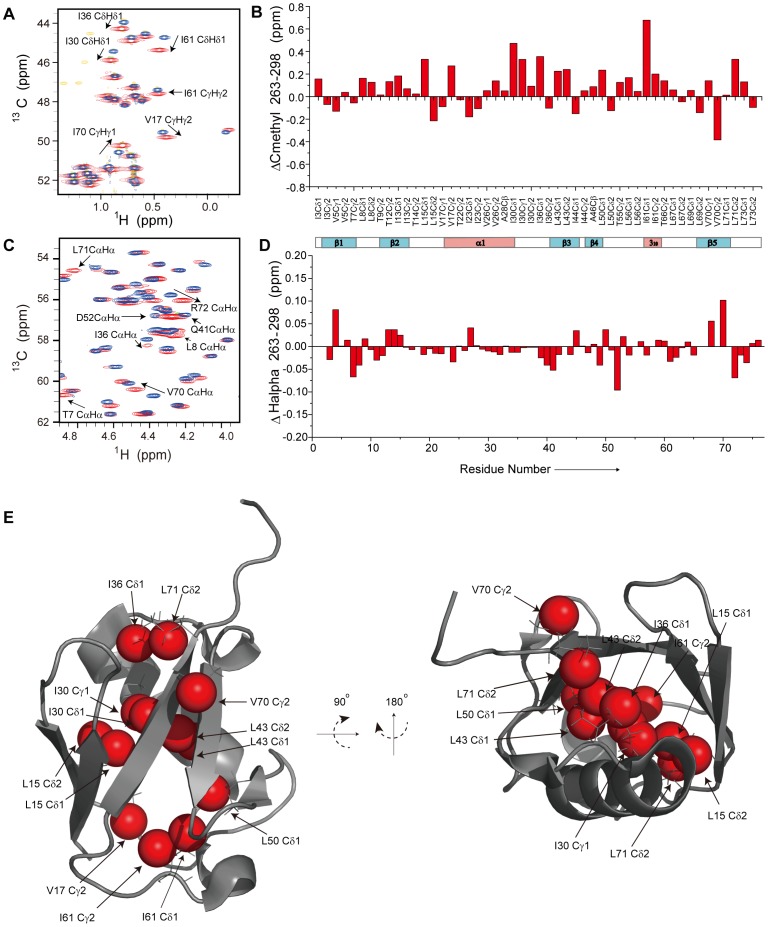
Cold-induced perturbation of the hydrophobic core of ubiquitin. Superposition of the methyl (A) and backbone Cα (C) regions of 2D [^1^H,^13^C] constant-time HSQC spectra at 263 K (red for positive peaks and green for negative peaks) and 298 K (blue for positive peaks and yellow for negative peaks). (D) Differences in Hα chemical shifts at 263 K and 298 K as a function of residue number in ubiquitin. The location of helices and β-strands is schematically shown above. (E) Methyl carbon atoms that experience chemical shift changes of more than 0.2 ppm when going from 298 K to 263 K (see B)) are highlighted on the 3D structure of ubiquitin.

To probe for structural changes connected to the weakening of hydrogen bonds, we recorded residual dipolar couplings. Residual dipolar couplings depend on the orientation of internuclear vectors relative to the magnetic field and are therefore highly sensitive reporters of protein structure [23]. [^1^H,^15^N] residual dipolar couplings (^1^D_NH_) measured at 298 K, 278 K, 270 K and 263 K fit very well to the 3D structure of ubiquitin that was determined at 298 K (PDB code: 1D3Z; Figures 4 and S3). The data demonstrate that the protein is folded down to 263 K in supercooled solution, in agreement with previous observations that ubiquitin is thermodynamically stable at least down to 241 K [11]. At subzero temperatures the quality of the fit decreased as a result of the lower experimental signal-to-noise ratio in the spectra, which is due to the much slower tumbling at low temperature (Figure S4) [9], such that at 263 K none of the experimental [^1^H, ^15^N] residual dipolar coupling deviated significantly from the values expected on the basis of the native structure (Figure S3A). At 270 K the dipolar couplings for a few residues (Glu34, Glu64, Ala46, Ile61) slightly deviated from the native structure (Figure 4A), but repeat measurements indicated larger experimental errors than estimated on the basis of the signal-to-noise ratio for Ala46 and Glu64 (Figure S3C). The observation that the ^1^D_NH_ value observed for Glu34 does not fit to the published 3D structure suggests that this residue might indeed experience slight structural changes at decreasing temperatures and in line with the non-linearity of its chemical shift changes (Figures 2A,B).

**Figure 6:**
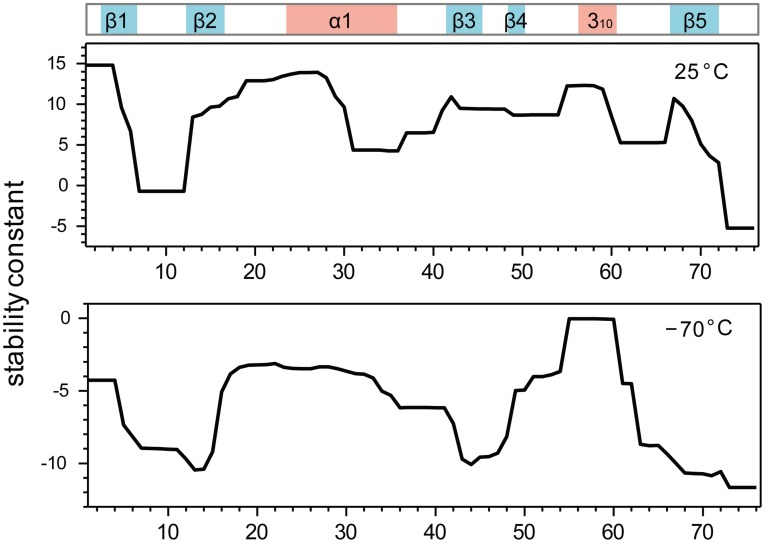
Stability constants of ubiquitin at 298 K and 203 K calculated by the COREX/BEST server. The location of helices and β-strands in the native state is schematically shown above. To reveal regions most prone to cold-denaturation, the calculations were performed at 203 K, i.e. below the temperature of cold-denaturation of ubiquitin.

To obtain further insight into the impact of low temperature on the 3D structure of ubiquitin, we measured two-dimensional [^1^H,^13^C]-HSQC spectra (Figure 5). Down to 263 K only very small chemical shift changes were observed. Cα and Hα secondary chemical shifts observed at 263 K were highly similar to the values at 298 K (Figures 5D, S5A and Table S2). We could not detect a consistent increase or decrease of secondary chemical shifts, with the exception of the Cα secondary chemical shifts in the α-helix of ubiquitin that were slightly increased at 263 K (Figure S5A). The largest changes in Hα chemical shifts were observed in the regions comprising Leu8 and Val70 (Figure 5D). Analysis of the methyl groups showed that all side chains pointing into the core of the ubiquitin structure were affected (Figures 5B,E and Table S3). The strongest changes in methyl proton chemical shifts were observed for Val17, Ile61, Leu69 and Val70 (Figures S5B,C). The data suggest that decreasing temperatures result in conformational rearrangements in the hydrophobic core of ubiquitin.

**Figure 7:**
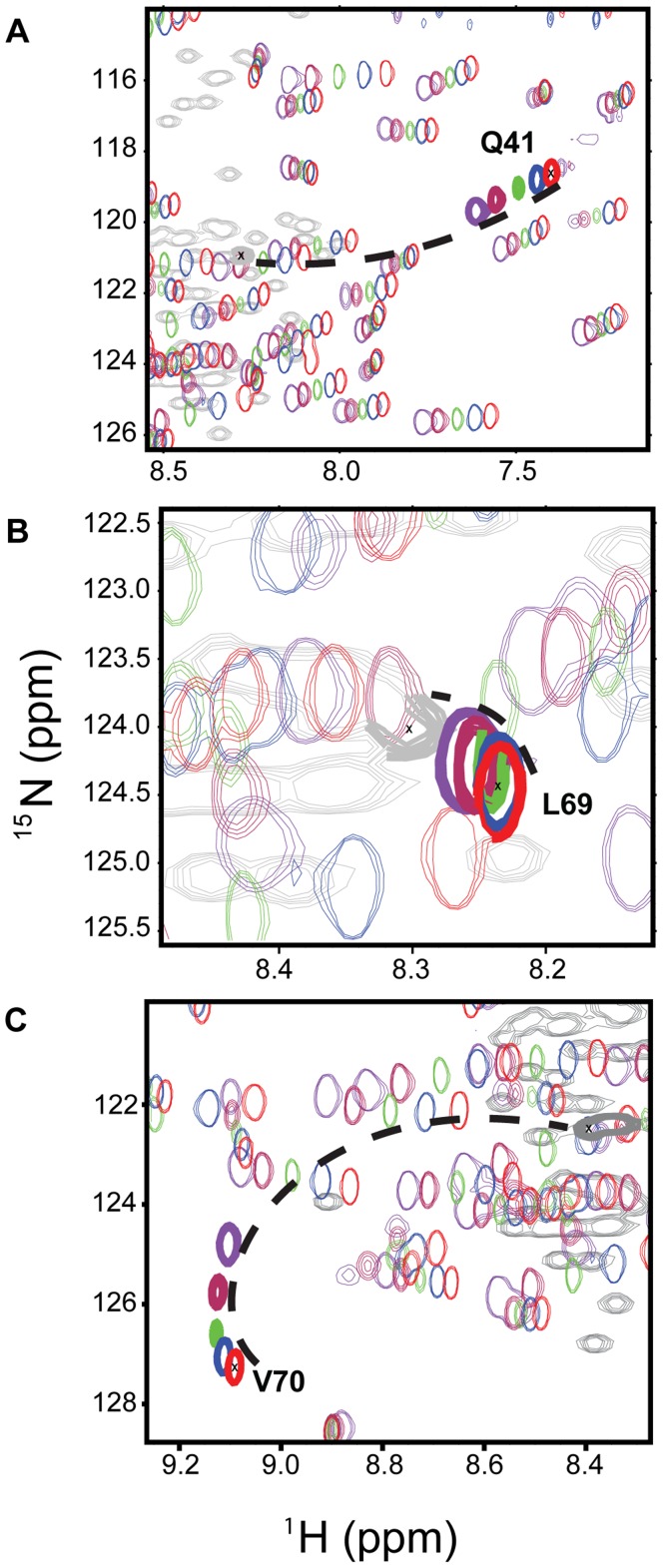
^1^H-^15^N chemical shift changes of specific residues in comparison to the chemically denatured state. Selected regions of 2D [^1^H,^15^N]-HSQCs with decreasing temperature: 298 K (red), 288 K (blue), 278 K (green), 268 K (maroon), and 260 K (purple). The unfolded state of ubiquitin was obtained by addition of 8 M urea at pH 2 (shown in thick grey).

Previously, it was demonstrated that ubiquitin encapsulated in reverse micelles undergoes cold-denaturation at about 253 K [24]. Residues in spatial proximity to Val70 were the ones that were most affected at the onset of cold-denaturation [24]. Figure 2C demonstrates that the same regions showed non-linear chemical shift changes and that these changes can be connected to a weakening of the corresponding backbone hydrogen bonds (Figure 3B). A lower stability of these regions is also suggested by residue-specific stability constants (Figure 6) that were estimated on the basis of the 3D structure of ubiquitin using the COREX algorithm [24], [25], [26]. Note that we performed on purpose – that is to investigate cold-denaturation of ubiquitin - the calculations at 203 K, as 203 K is below the temperature at which ubiquitin cold-denatures in solution [11]. At 263 K ubiquitin showed only small changes in the regional variations in stability (data not shown), in line with its folded state (Figure 4). In contrast, at 203 K COREX predicts reduced stability for residues 8, 13, 42–44 and 68–70. Another hint that the observed non-linear chemical shift changes and the weakening of hydrogen bonds (Figures 1, 2 and 3) might be related to cold-denaturation comes from a comparison with NMR chemical shifts of chemically denatured ubiquitin (Figure 7): some cross peaks in the [^1^H,^15^N]-HSQC spectra appear to move towards the unfolded state of ubiquitin in 8 M urea at pH 2 (BMRB 4375) [27]. However, for other residues such as Leu8 and Ile44 no such trend was apparent, either because there is no connection or it cannot be established because of the largely different sample conditions that is pH 2 and 8 M urea in the chemically denatured state.

A certain amount of conformational plasticity is important to accommodate a binding partner at the binding site. Here we compared the number of contacts for each residue of ubiquitin involved in known ubiquitin-protein complex structures (Figure 2A, red) [4] to the profile of significant changes in amide proton chemical shifts (Figure 2A, black bar): a good match between residues affected by low temperature in supercooled solution and those involved in intermolecular interactions was observed. We further extended the comparison to the intrinsic dynamics of the backbone of ubiquitin (Figure 2A, green). To this end we used the amide ^1^H-^15^N differential relaxation rate constants for zero and double quantum coherences that were previously reported by Palmer and co-workers [28], as these rates highlight residues that experience slow time scale dynamics. The dynamic residues of ubiquitin can be grouped into two clusters. Cluster one is the ubiquitin binding surface containing Leu8, Ile44 and Val70 that is affected by the transition into supercooled solution (Figures 1 and 2). The second cluster comprises Ile23 and Thr55 connecting the N-terminus of the α-helix and the loop residues Glu51-Leu56. At pH 7.0, the pH of our measurement, Glu24 and Gly53 were not observed at any temperature due to strong line broadening. The slow motions in this region have recently been attributed to a hydrogen bond between the side-chain carboxyl oxygen of Glu24 and its backbone amide, as well as the amide of Gly53 [29]. This hydrogen bond was suggested to regulate slow motions in ubiquitin by modulating a β-turn flip at residues Glu51 through Arg54 [29]. The comparison shown in Figure 2A indicates that the dynamic cluster comprising I23 and T55 is not strongly perturbed in supercooled solution and is also not at the core of the binding hot spot of ubiquitin. In contrast, a good match was present between regions in which the hydrogen bonds are weakened in supercooled solution and those involved in protein-protein interactions.

Our data demonstrate that at low temperature in supercooled solution the 3D structure of ubiquitin is perturbed. This perturbation is very gentle with only minor changes in chemical shifts - that is the state of ubiquitin at 263 K is not a partially denatured state and the observed changes in chemical shifts may well reflect local changes in structure unrelated to cold-denaturation. However, trans-hydrogen bond scalar couplings demonstrated that the hydrogen bonds in the β-sheet of ubiquitin are already weakened at 270 K. Thus, the enhanced motions that were previously detected for Val70 and Ile13 in supercooled water [5], [30] might be related to the weakening of hydrogen bonds in supercooled solution. In addition, the region where we find changes in trans-hydrogen bond scalar couplings as well as the non-linear behavior of amide proton chemical shifts overlaps with the region that is most sensitive to cold-denaturation in reverse micelles [24]. Thus, the weakening of the hydrogen bonds that we observed in this region already at 270 K might be one of the first events that occur during cold-denaturation of ubiquitin. The observation that it is also the region that is most strongly involved in protein-protein interaction is in line with the hypothesis that this part of ubiquitin more easily adjusts to the conformational changes that are required for complex formation.

## Materials and Methods

^15^N and ^13^C/^15^N-labeled ubiquitin was prepared as described previously [4]. NMR samples contained 0.5 mM of protein in 50 mM HEPES, pH 7, 300 mM NaCl. The use of HEPES results in a small pH increase as the temperature decreases (−0.014/°C), so that the overall pH change is below 0.5, which results in only very weak changes in the HSQC spectrum of ubiquitin. Chemical shift referencing was done with an additional capillary tube containing 0.1% DSS dissolved in the same buffer solution.

NMR spectra were acquired on a Bruker 700 MHz NMR spectrometer. To avoid freezing at sub-zero temperatures, the protein solution was put into glass capillaries of 1.0 mm outer diameter, and the capillaries were placed in a 5 mm NMR tube [9]. Two-dimensional [^1^H,^15^N]-HSQC spectra were recorded at 298 K, 293 K, 288 K, 284 K, 280 K, 278 K, 276.5 K, 274.5 K, 274 K, 273.5 K, 273.2 K, 272.7 K, 272 K, 271 K, 270 K, 269 K, 268 K, 266 K, 265 K and 263 K. Temperatures were carefully adjusted to the desired value and checked by a methanol reference sample. NMR data were processed and analyzed using NMRPipe [31] and Sparky 3 (University of California, San Francisco). The weighted average of ^1^H and ^15^N chemical shift changes was calculated according to ((ΔH)^2^+(ΔN/5)^2^)^½^ and fitted to a linear function of temperature. For the comparison of two linear fittings above or below a specific temperature, the slope comparison function [32] in PRISM (GraphPad, CA) was used. The change of slope (Diff/Ref in Table S1) was calculated as the difference of the two slopes divided by the slope for the 298 K-273 K temperature range. Two-dimensional constant-time [^1^H,^13^C]-HSQC spectra were recorded at 298 K, 278 K, 270 K, 268 K and 263 K with 512×125 complex points (sweep width = 30 ppm, carrier at 54 ppm). Side-chain and backbone assignments of ubiquitin at 298 K were taken from the BMRB database (code 6457) and were transferred to lower temperatures by following the temperature-dependent chemical shift changes.

[^1^H,^15^N] residual dipolar couplings (^1^D_NH_) were determined by using the two-dimensional inphase-antiphase (IPAP)-HSQC sequence [33]. D_NH_ values were calculated as the difference between splittings measured in the isotropic phase and in a sample, in which ubiquitin had been aligned in 10 mg/ml Pf1 bacteriophage (Asla, Riga, Latvia). RDCs were not corrected for the negative gyromagnetic ratio of ^15^N. Errors in experimental RDCs were calculated based on signal-to-noise ratios [34]. RDCs were best-fit to the 3D structure of ubiquitin using singular value decomposition as implemented in the software PALES [35]. Errors in back-calculated RDCs were obtained by performing the singular value decomposition for all members of the ensemble of NMR structures (PDB code: 1D3Z).

^h3^J_NC’_ trans-hydrogen bond measurements were carried on a sample of 5 mM ^2^H/^15^N/^13^C-labeled ubiquitin with deuteration lever higher than 80%, in 50 mM phosphate buffer, pH 6.5. The trans H-bond scalar couplings were measured by 2D long-range Trosy-HNCO experiments as described previously [20], [21], [22]. Acquisition times on carbonyl and amide proton were 48.3 ms and 56.3 ms, respectively. Reference spectra were recorded in 5.2 h at 270 K, 1.8 h at 278 K and 298 K. Cross peak spectra required 17.3 h at 298 K, 43.2 h at 278 K, 64 hours at 270 k, respectively, to obtain high signal-to-noise ratios.

COREX calculations were performed using the COREX/BEST online server at http://best.bio.jhu.edu/BEST/ [26]. Model 1 of the NMR ensemble (PDB code: 1D3Z) was used as template. Calculations were performed as described previously [6].

## Supporting Information

Figure S1 **Weighted average of backbone ^1^H, ^15^N chemical shift changes for all non-overlapping, non-proline residues in ubiquitin induced by cooling down from 298 K to 263 K.** Red lines indicate straight-line fits for the range 298 K-273 K. Dashed grey lines indicate 273 K.
(TIF)

Figure S2 **Amide proton (upper) and amide nitrogen (lower) temperature coefficients of ubiquitin within 298 K-273 K (grey bars) and 273 K-263 K (blue line).** Amide protons with temperature coefficients of less than −4.6 ppb/K are likely to be not involved in hydrogen bonds [17].
(TIF)

Figure S3 **[^1^H,^15^N] residual dipolar couplings at decreasing temperatures.** Correlation between experimental ^1^H–^15^N RDCs at (A) 263 K and (B) at 298 K to couplings calculated from the best-fit to the solution NMR structure of ubiquitin (PDB entry 1D3Z) using singular value decomposition. (C) Correlation of RDC values from two independent measurements at 270 K. (D) RDC quality factor of the best-fit of RDCs to the solution structure of ubiquitin at different temperatures. Lower RDC quality factors indicate a better fit to the structure. The increase in RDC quality factor at lower temperatures is most likely due to the lower signal-to-noise ratio of the NMR spectra of ubiquitin at low temperatures that is caused by the slower overall tumbling (see Figure S4).
(TIF)

Figure S4 **Global rotational correlation times of ubiquitin for decreasing temperatures.** Global correlation times were estimated from ^1^H–^15^N TRACT experiments^2^. The global correlation time is due to the increase in viscosity at low temperatures and can be predicted from hydrodynamic theory as reported previously for ubiquitin [9].
(TIF)

Figure S5 **Chemical shifts changes at low temperature in supercooled solution.** (A) Cα chemical shift differences between 263 K and 298 K as a function of residue number in ubiquitin. The location of helices and β-strands is schematically shown above. (B) Methyl proton chemical shifts difference between 263 K and 298 K. (C) Methyl protons that experience chemical shift changes of more than 0.02 ppm when going from 298 K to 263 K (see B)) are highlighted on the 3D structure of ubiquitin.
(TIF)

Table S1 **^1^H and ^15^N temperature coefficients of ubiquitin and statistical analysis for two linear regressions on 298-273 K vs. 273-263 K.**
(DOC)

Table S2 **Cα and Hα Chemical shifts of ubiquitin at 298 K, 278 K and 263 K.**
(DOC)

Table S3 **Carbon and Proton Chemical shifts of ubiquitin methyl groups at 298 K, 278 K and 263 K.**
(DOC)
