# Supplementary figures and images for: Cold-Induced Changes in the Protein Ubiquitin
Source: PLoS One. 2012 Jun 21;7(6):e37270. doi: 10.1371/journal.pone.0037270 (PMC3380907; doi:10.1371/journal.pone.0037270)

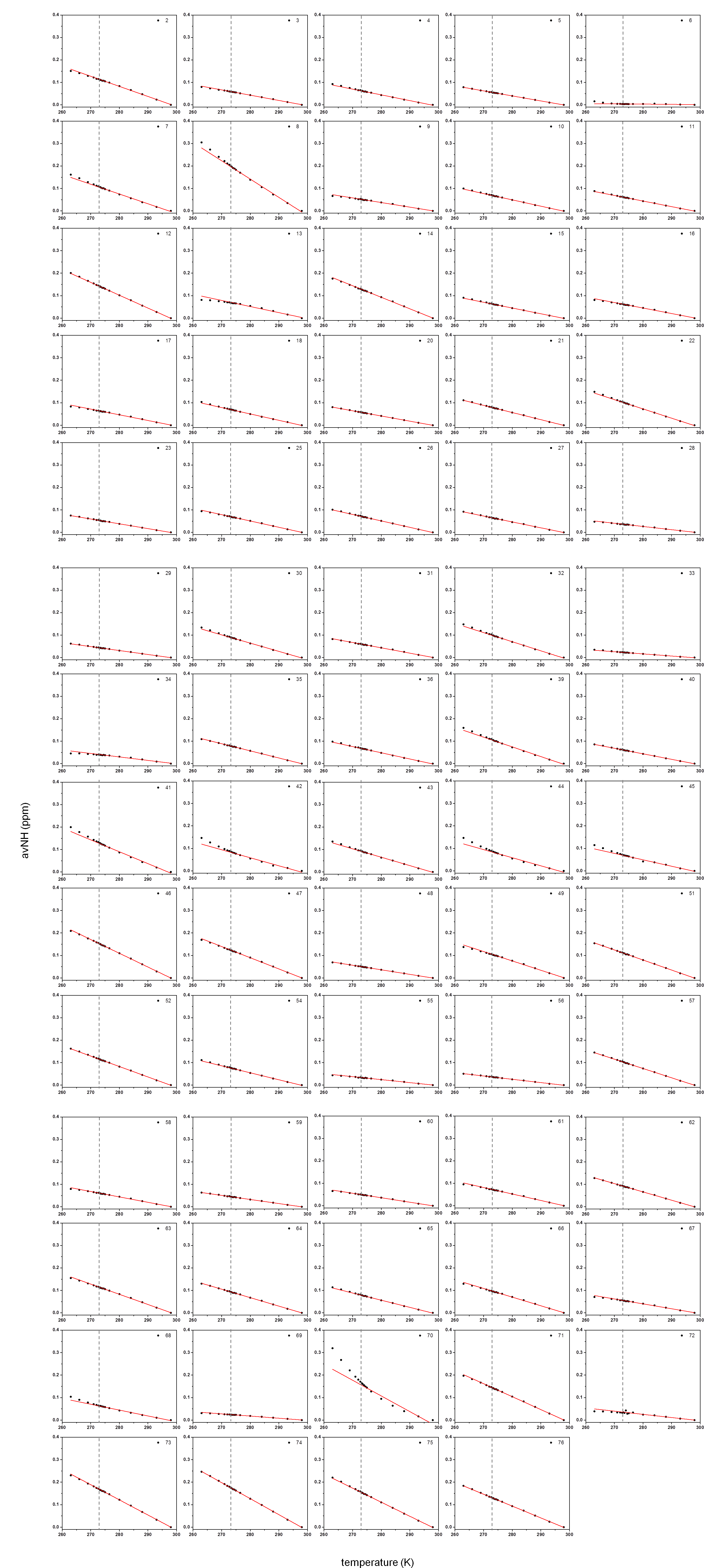

Supplement: Figure S1 — Weighted average of backbone 1H, 15N chemical shift changes for all non-overlapping, non-proline residues in ubiquitin induced by cooling down from 298 K to 263 K. Red lines indicate straight-line fits for the range 298 K-273 K. Dashed grey lines indicate 273 K. (TIF) [file pone.0037270.s001.tif]

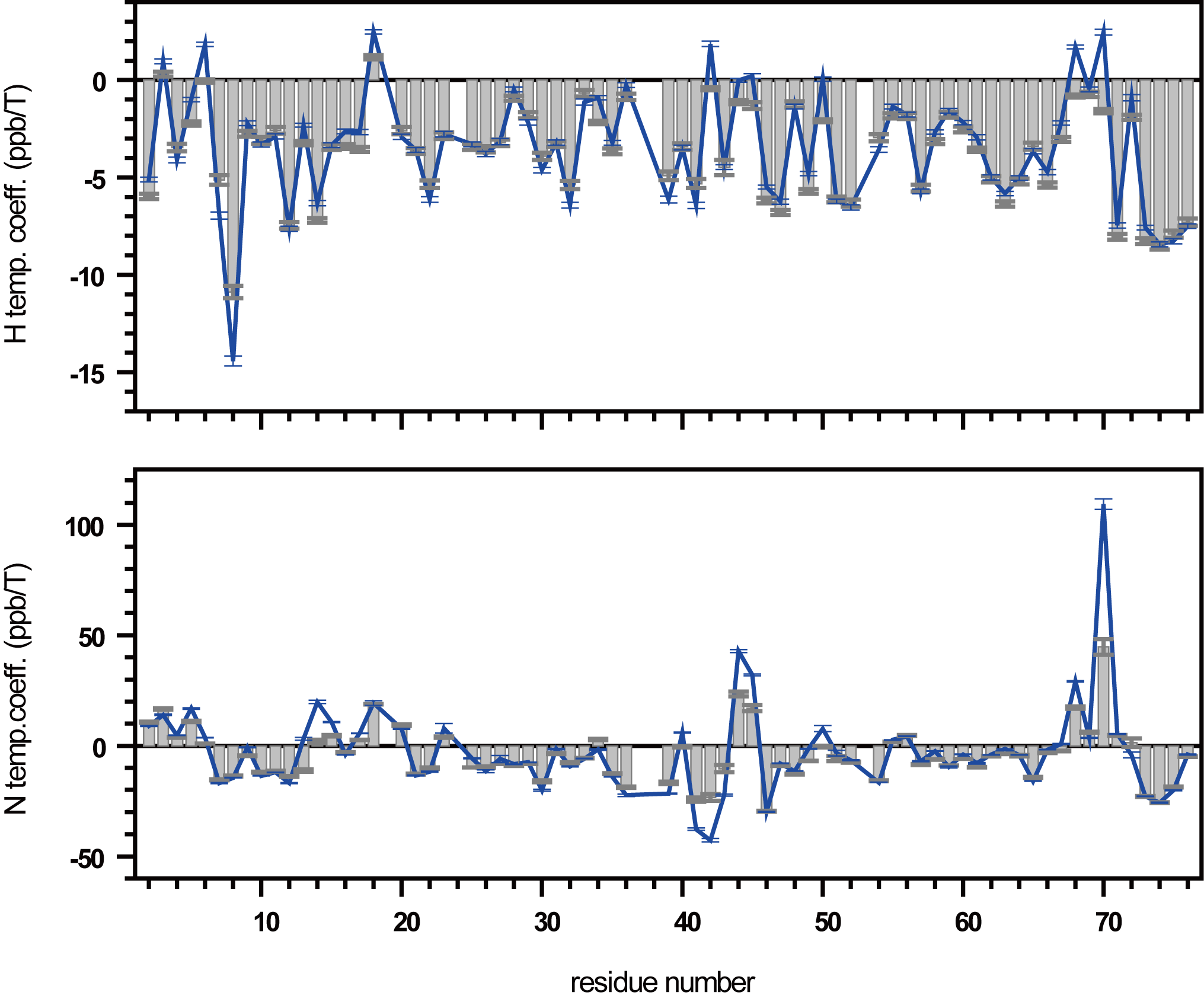

Supplement: Figure S2 — Amide proton (upper) and amide nitrogen (lower) temperature coefficients of ubiquitin within 298 K-273 K (grey bars) and 273 K-263 K (blue line). Amide protons with temperature coefficients of less than −4.6 ppb/K are likely to be not involved in hydrogen bonds [17]. (TIF) [file pone.0037270.s002.tif]

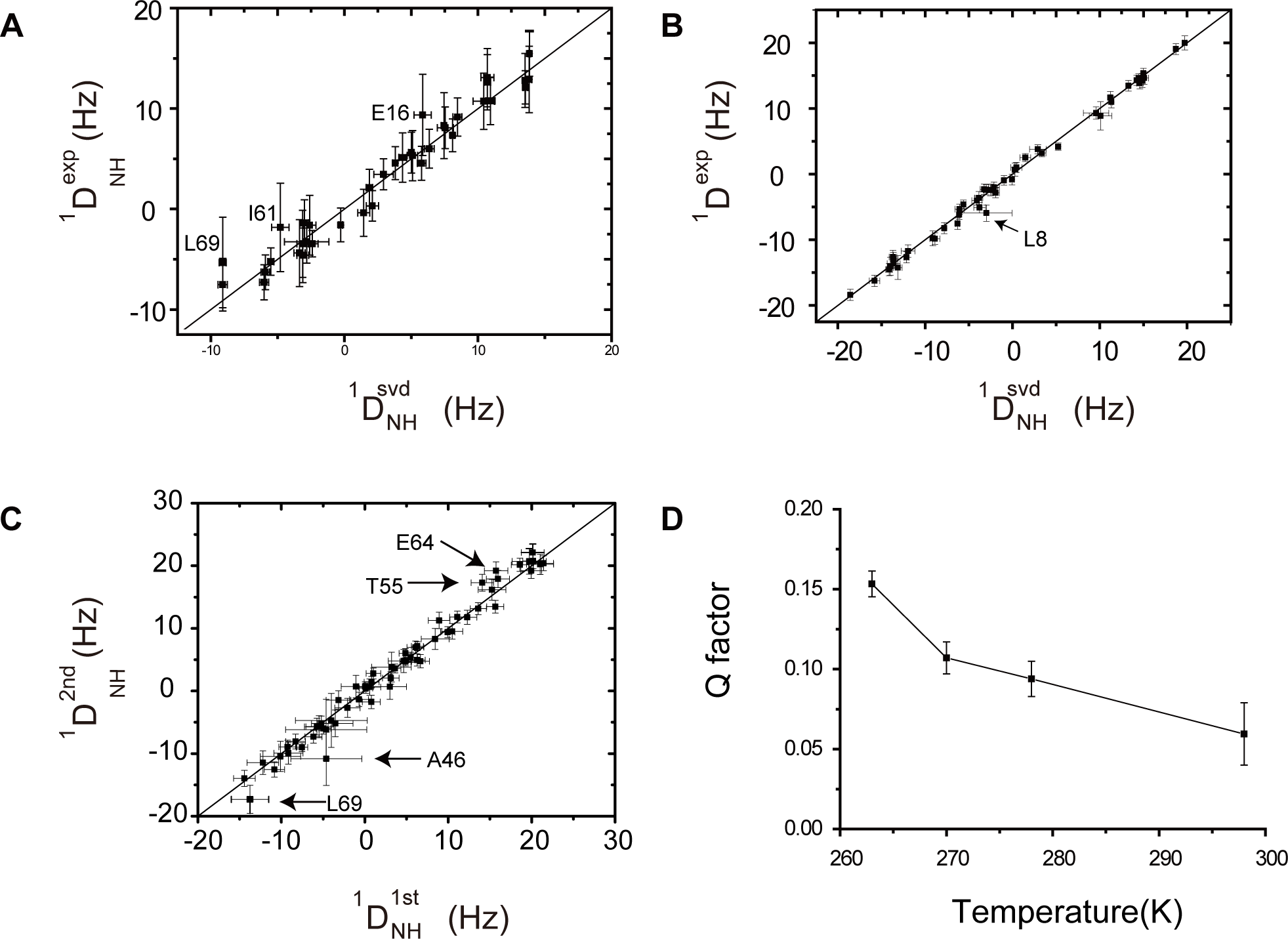

Supplement: Figure S3 — [1H,15N] residual dipolar couplings at decreasing temperatures. Correlation between experimental 1H–15N RDCs at (A) 263 K and (B) at 298 K to couplings calculated from the best-fit to the solution NMR structure of ubiquitin (PDB entry 1D3Z) using singular value decomposition. (C) Correlation of RDC values from two independent measurements at 270 K. (D) RDC quality factor of the best-fit of RDCs to the solution structure of ubiquitin at different temperatures. Lower RDC quality factors indicate a better fit to the structure. The increase in RDC quality factor at lower temperatures is most likely due to the lower signal-to-noise ratio of the NMR spectra of ubiquitin at low temperatures that is caused by the slower overall tumbling (see Figure S4). (TIF) [file pone.0037270.s003.tif]

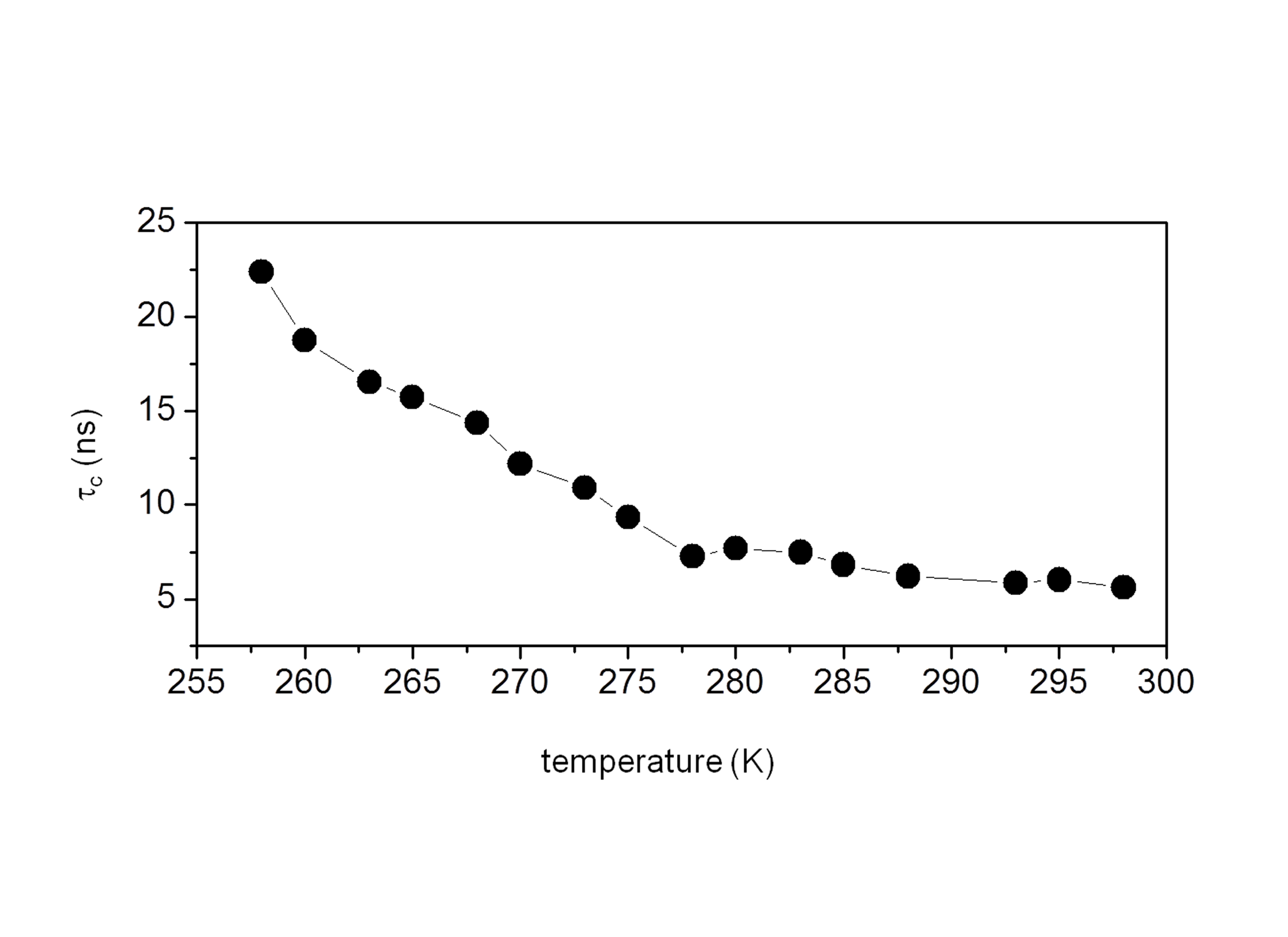

Supplement: Figure S4 — Global rotational correlation times of ubiquitin for decreasing temperatures. Global correlation times were estimated from 1H–15N TRACT experiments2. The global correlation time is due to the increase in viscosity at low temperatures and can be predicted from hydrodynamic theory as reported previously for ubiquitin [9]. (TIF) [file pone.0037270.s004.tif]

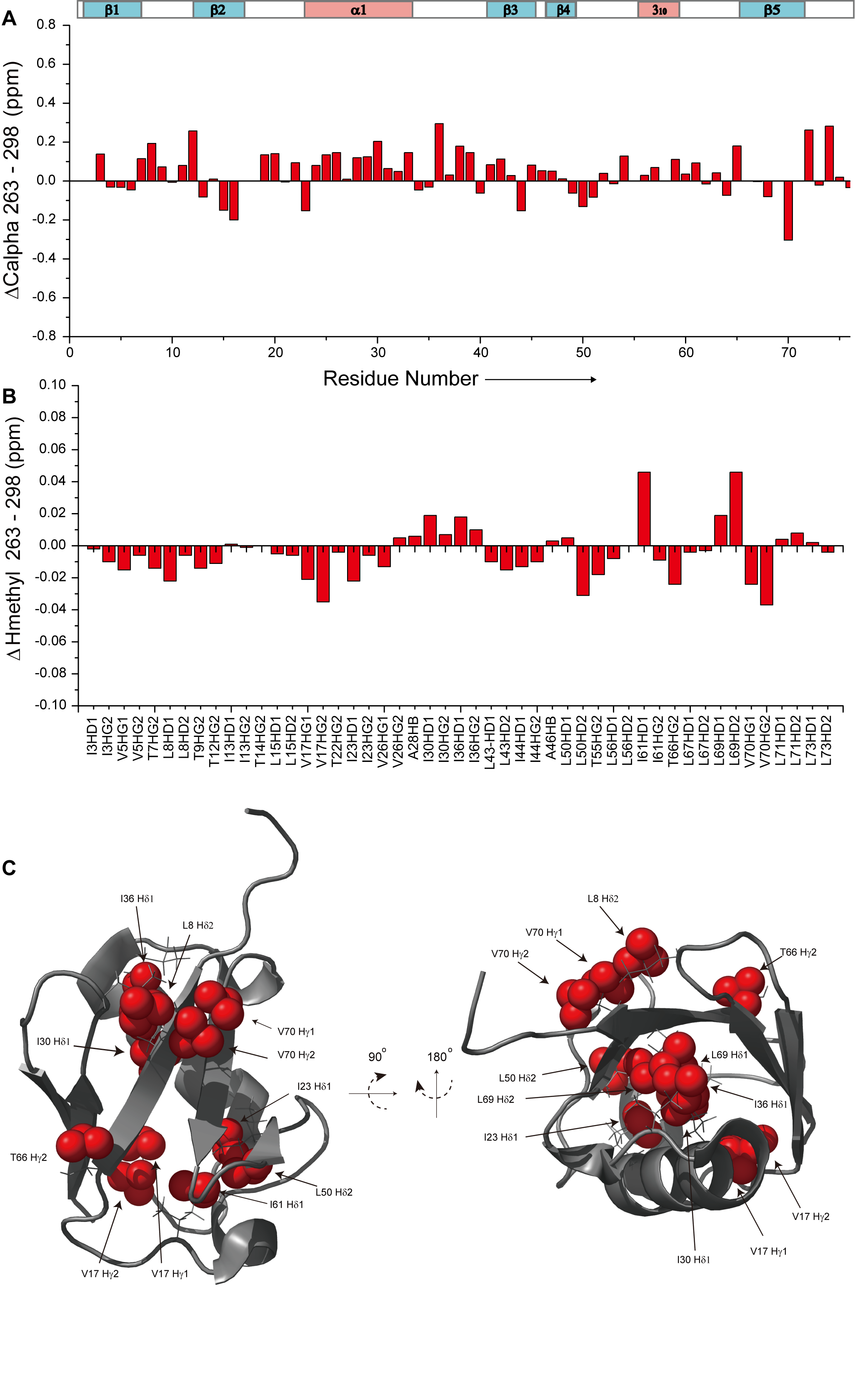

Supplement: Figure S5 — Chemical shifts changes at low temperature in supercooled solution. (A) Cα chemical shift differences between 263 K and 298 K as a function of residue number in ubiquitin. The location of helices and β-strands is schematically shown above. (B) Methyl proton chemical shifts difference between 263 K and 298 K. (C) Methyl protons that experience chemical shift changes of more than 0.02 ppm when going from 298 K to 263 K (see B)) are highlighted on the 3D structure of ubiquitin. (TIF) [file pone.0037270.s005.tif]
