# Supplementary material for: Cold-Induced Changes in the Protein Ubiquitin
Source: PLoS One. 2012 Jun 21;7(6):e37270. doi: 10.1371/journal.pone.0037270 (PMC3380907; doi:10.1371/journal.pone.0037270)
Supplement: Table S1 — 1H and 15N temperature coefficients of ubiquitin and statistical analysis for two linear regressions on 298-273 K vs. 273-263 K. (DOC) [file pone.0037270.s006.doc]

**Table S1. ^1^H and ^15^N temperature coefficients of ubiquitin and statistical analysis for two linear regressions on 298-273K vs. 273-263K.**

| Residue Number | H temperature coefficient (ppb/K) | | |  | N temperature coefficient (ppb/K) | | |  | Slope comparison | | | |
| --- | --- | --- | --- | --- | --- | --- | --- | --- | --- | --- | --- | --- |
|  | 298-273K | 273-263K | Difference |  | 298-273K | 273-263K | Difference |  | H | | N | |
|  |  |  |  |  |  |  |  |  | F value | P value | F value | P value |
| 2 | -5.97 | -5.10 | 0.87 |  | 10.72 | 8.96 | -1.76 |  | 9.18 | 3.27E-03 | 21.81 | 7.02E-05 |
| 3 | 0.31 | 0.96 | 0.65 |  | 16.58 | 14.02 | -2.56 |  | 8.10 | 5.19E-03 | 15.83 | 3.29E-04 |
| 4 | -3.46 | -4.11 | -0.65 |  | 3.82 | 4.77 | 0.95 |  | 7.37 | 7.25E-03 | 12.09 | 1.08E-03 |
| 5 | -2.23 | -1.01 | 1.23 |  | 11.01 | 16.91 | 5.91 |  | 22.06 | 7.00E-05 | 24.39 | 3.98E-05 |
| 6 | -0.06 | 1.84 | 1.90 |  | 1.01 | 3.75 | 2.75 |  | 24.38 | 4.00E-05 | 36.06 | 4.96E-06 |
| 7 | -5.13 | -6.95 | -1.82 |  | -15.34 | -16.92 | -1.59 |  | 18.68 | 1.50E-04 | 4.75 | 2.83E-02 |
| 8 | -10.88 | -14.42 | -3.54 |  | -13.58 | -14.42 | -0.85 |  | 20.83 | 9.00E-05 | 1.70 | 2.20E-01 |
| 9 | -2.75 | -2.22 | 0.54 |  | -4.51 | -0.67 | 3.84 |  | 9.48 | 2.89E-03 | 38.83 | 3.29E-06 |
| 10 | -3.11 | -3.31 | -0.21 |  | -11.86 | -13.54 | -1.68 |  | 2.64 | 1.09E-01 | 8.92 | 3.64E-03 |
| 11 | -2.59 | -2.89 | -0.31 |  | -11.47 | -11.95 | -0.49 |  | 4.19 | 3.94E-02 | 0.80 | 4.72E-01 |
| 12 | -7.46 | -7.64 | -0.18 |  | -13.86 | -16.82 | -2.96 |  | 2.08 | 1.65E-01 | 11.28 | 1.45E-03 |
| 13 | -3.23 | -2.32 | 0.91 |  | -11.17 | 3.14 | 14.31 |  | 19.22 | 1.30E-04 | 41.85 | 2.16E-06 |
| 14 | -7.21 | -6.32 | 0.89 |  | 2.01 | 19.83 | 17.83 |  | 11.61 | 1.28E-03 | 39.38 | 3.04E-06 |
| 15 | -3.45 | -3.35 | 0.10 |  | 4.59 | 10.67 | 6.08 |  | 2.40 | 1.29E-01 | 30.74 | 1.18E-05 |
| 16 | -3.42 | -2.63 | 0.78 |  | -3.11 | -4.01 | -0.90 |  | 14.51 | 4.90E-04 | 23.57 | 4.74E-05 |
| 17 | -3.56 | -2.67 | 0.89 |  | 2.62 | 5.68 | 3.07 |  | 13.44 | 6.90E-04 | 19.85 | 1.12E-04 |
| 18 | 1.18 | 2.47 | 1.29 |  | 18.82 | 19.34 | 0.52 |  | 14.71 | 4.60E-04 | 2.61 | 1.12E-01 |
| 20 | -2.60 | -2.92 | -0.32 |  | 9.37 | 7.64 | -1.74 |  | 6.04 | 1.40E-02 | 16.18 | 2.97E-04 |
| 21 | -3.64 | -3.58 | 0.06 |  | -12.77 | -13.47 | -0.70 |  | 1.48 | 2.63E-01 | 4.42 | 3.43E-02 |
| 22 | -5.35 | -6.14 | -0.79 |  | -10.10 | -11.74 | -1.65 |  | 9.38 | 3.01E-03 | 8.51 | 4.33E-03 |
| 23 | -2.85 | -2.78 | 0.07 |  | 3.86 | 7.99 | 4.13 |  | 2.24 | 1.45E-01 | 12.85 | 8.33E-04 |
| 25 | -3.42 | -3.30 | 0.13 |  | -9.78 | -5.64 | 4.14 |  | 3.63 | 5.61E-02 | 45.29 | 1.38E-06 |
| 26 | -3.55 | -3.80 | -0.25 |  | -9.65 | -11.47 | -1.82 |  | 5.23 | 2.16E-02 | 5.61 | 1.75E-02 |
| 27 | -3.21 | -3.15 | 0.06 |  | -8.29 | -5.95 | 2.34 |  | 2.46 | 1.24E-01 | 0.69 | 5.20E-01 |
| 28 | -0.93 | -0.48 | 0.44 |  | -8.98 | -8.35 | 0.63 |  | 5.90 | 1.50E-02 | 4.46 | 3.36E-02 |
| 29 | -1.82 | -2.18 | -0.36 |  | -8.10 | -7.50 | 0.60 |  | 9.50 | 2.86E-03 | 8.05 | 5.31E-03 |
| 30 | -3.91 | -4.62 | -0.71 |  | -16.01 | -20.13 | -4.12 |  | 9.06 | 3.43E-03 | 14.87 | 4.37E-04 |
| 31 | -3.29 | -3.22 | 0.07 |  | -3.46 | -1.39 | 2.07 |  | 0.89 | 4.33E-01 | 10.25 | 2.13E-03 |
| 32 | -5.39 | -6.44 | -1.05 |  | -7.58 | -9.31 | -1.73 |  | 12.76 | 8.60E-04 | 32.63 | 8.56E-06 |
| 33 | -0.68 | -1.13 | -0.45 |  | -5.52 | -5.81 | -0.29 |  | 12.87 | 8.30E-04 | 1.28 | 3.11E-01 |
| 34 | -2.18 | -0.90 | 1.28 |  | 2.91 | -1.63 | -4.54 |  | 20.46 | 1.00E-04 | 51.18 | 6.87E-07 |
| 35 | -3.66 | -3.23 | 0.43 |  | -12.53 | -14.02 | -1.49 |  | 6.92 | 9.00E-03 | 6.32 | 1.21E-02 |
| 36 | -0.85 | -0.26 | 0.59 |  | -18.73 | -22.34 | -3.61 |  | 20.69 | 9.00E-05 | 9.98 | 2.36E-03 |
| 39 | -4.90 | -6.13 | -1.23 |  | -16.77 | -21.65 | -4.88 |  | 13.55 | 6.60E-04 | 13.49 | 6.75E-04 |
| 40 | -3.42 | -3.47 | -0.04 |  | -0.55 | 5.96 | 6.51 |  | 2.12 | 1.59E-01 | 30.12 | 1.32E-05 |
| 41 | -5.32 | -6.44 | -1.12 |  | -24.37 | -37.73 | -13.37 |  | 12.54 | 9.30E-04 | 21.87 | 6.92E-05 |
| 42 | -0.44 | 1.86 | 2.30 |  | -23.40 | -42.67 | -19.27 |  | 23.05 | 5.00E-05 | 22.53 | 5.96E-05 |
| 43 | -4.49 | -4.47 | 0.03 |  | -10.32 | -22.31 | -11.99 |  | 2.46 | 1.24E-01 | 25.85 | 2.95E-05 |
| 44 | -1.13 | -0.01 | 1.12 |  | 23.49 | 42.94 | 19.45 |  | 19.02 | 1.40E-04 | 24.25 | 4.10E-05 |
| 45 | -1.31 | 0.20 | 1.51 |  | 17.08 | 32.12 | 15.04 |  | 21.79 | 7.00E-05 | 21.47 | 7.59E-05 |
| 46 | -6.18 | -5.49 | 0.69 |  | -29.57 | -29.74 | -0.17 |  | 11.43 | 1.37E-03 | 1.71 | 2.20E-01 |
| 47 | -6.80 | -6.24 | 0.56 |  | -9.01 | -8.01 | 1.01 |  | 6.39 | 1.17E-02 | 13.33 | 7.10E-04 |
| 48 | -1.25 | -1.36 | -0.12 |  | -12.79 | -11.41 | 1.38 |  | 2.74 | 1.02E-01 | 12.16 | 1.05E-03 |
| 49 | -5.71 | -4.81 | 0.91 |  | -6.81 | -1.37 | 5.44 |  | 15.24 | 3.90E-04 | 53.68 | 5.22E-07 |
| 51 | -6.14 | -6.22 | -0.08 |  | -5.74 | -3.28 | 2.47 |  | 1.50 | 2.60E-01 | 0.59 | 5.67E-01 |
| 52 | -6.32 | -6.54 | -0.22 |  | -7.34 | -6.55 | 0.79 |  | 2.51 | 1.20E-01 | 8.71 | 3.98E-03 |
| 54 | -2.96 | -3.57 | -0.60 |  | -15.74 | -16.59 | -0.84 |  | 12.17 | 1.05E-03 | 2.05 | 1.69E-01 |
| 55 | -1.83 | -1.36 | 0.47 |  | 1.78 | 2.68 | 0.90 |  | 5.49 | 1.87E-02 | 6.40 | 1.16E-02 |
| 56 | -1.85 | -1.77 | 0.07 |  | 4.64 | 4.31 | -0.33 |  | 1.98 | 1.78E-01 | 5.99 | 1.44E-02 |
| 57 | -5.54 | -5.72 | -0.18 |  | -8.48 | -6.92 | 1.55 |  | 3.14 | 7.70E-02 | 6.81 | 9.47E-03 |
| 58 | -3.16 | -2.67 | 0.49 |  | -6.27 | -2.32 | 3.95 |  | 9.40 | 2.99E-03 | 31.90 | 9.67E-06 |
| 59 | -1.78 | -1.58 | 0.20 |  | -8.93 | -9.51 | -0.59 |  | 1.56 | 2.47E-01 | 1.55 | 2.49E-01 |
| 60 | -2.53 | -2.17 | 0.36 |  | -5.87 | -3.91 | 1.97 |  | 4.98 | 2.48E-02 | 31.15 | 1.10E-05 |
| 61 | -3.58 | -2.94 | 0.63 |  | -9.63 | -7.89 | 1.73 |  | 8.92 | 3.65E-03 | 16.59 | 2.64E-04 |
| 62 | -5.09 | -5.00 | 0.10 |  | -4.60 | -4.02 | 0.58 |  | 2.36 | 1.34E-01 | 9.71 | 2.63E-03 |
| 63 | -6.36 | -5.82 | 0.54 |  | -3.54 | -1.34 | 2.20 |  | 6.76 | 9.69E-03 | 29.17 | 1.56E-05 |
| 64 | -5.21 | -5.03 | 0.18 |  | -4.45 | -4.10 | 0.36 |  | 2.61 | 1.12E-01 | 6.33 | 1.20E-02 |
| 65 | -3.39 | -3.66 | -0.27 |  | -14.33 | -15.95 | -1.62 |  | 4.14 | 4.06E-02 | 7.82 | 5.89E-03 |
| 66 | -5.39 | -4.72 | 0.67 |  | -3.22 | -1.72 | 1.50 |  | 8.91 | 3.66E-03 | 23.73 | 4.58E-05 |
| 67 | -3.06 | -2.20 | 0.85 |  | -2.29 | 0.75 | 3.04 |  | 14.35 | 5.10E-04 | 35.64 | 5.29E-06 |
| 68 | -0.83 | 1.71 | 2.53 |  | 17.33 | 29.17 | 11.84 |  | 33.43 | 1.00E-05 | 28.49 | 1.77E-05 |
| 69 | -0.74 | -0.49 | 0.25 |  | 6.10 | 3.60 | -2.50 |  | 3.39 | 6.55E-02 | 12.46 | 9.50E-04 |
| 70 | -1.59 | 2.47 | 4.06 |  | 44.73 | 109.29 | 64.56 |  | 32.67 | 1.00E-05 | 29.67 | 1.43E-05 |
| 71 | -8.04 | -7.47 | 0.58 |  | 4.62 | 5.33 | 0.70 |  | 6.21 | 1.28E-02 | 4.73 | 2.86E-02 |
| 72 | -1.91 | -0.91 | 1.00 |  | 1.44 | -4.15 | -5.60 |  | 6.44 | 1.14E-02 | 0.28 | 7.61E-01 |
| 73 | -8.26 | -7.59 | 0.68 |  | -22.84 | -22.82 | 0.03 |  | 7.92 | 5.64E-03 | 2.80 | 9.76E-02 |
| 74 | -8.51 | -8.43 | 0.08 |  | -25.71 | -25.79 | -0.08 |  | 2.97 | 8.65E-02 | 2.28 | 1.42E-01 |
| 75 | -7.90 | -8.27 | -0.36 |  | -18.63 | -20.17 | -1.54 |  | 3.24 | 7.23E-02 | 3.93 | 4.61E-02 |
| 76 | -7.30 | -7.50 | -0.20 |  | -4.87 | -3.91 | 0.95 |  | 2.04 | 1.70E-01 | 13.82 | 6.06E-04 |
