# Supplementary material for: Cold-Induced Changes in the Protein Ubiquitin
Source: PLoS One. 2012 Jun 21;7(6):e37270. doi: 10.1371/journal.pone.0037270 (PMC3380907; doi:10.1371/journal.pone.0037270)
Supplement: Table S2 — Cα and Hα Chemical shifts of ubiquitin at 298 K, 278 K and 263 K. (DOC) [file pone.0037270.s007.doc]

**Table S2. Cα and Hα Chemical shifts of ubiquitin at 298K, 278K and 263K.**

| Residue  number | Cα Chemical shifts(ppm) | | |  | Hα Chemical shifts(ppm) | | |
| --- | --- | --- | --- | --- | --- | --- | --- |
|  | 298K | 278K | 263K |  | 298K | 278K | 263K |
| 2 | 56.32 | 56.45 | N.A. |  | 5.13 | 5.10 | N.A. |
| 3 | 59.69 | 59.77 | 59.83 |  | 4.15 | 4.14 | 4.12 |
| 4 | 55.21 | 55.18 | 55.18 |  | 5.63 | 5.68 | 5.71 |
| 5 | 60.49 | 60.48 | 60.46 |  | 4.83 | 4.83 | 4.83 |
| 6 | 54.72 | 54.72 | 54.67 |  | 5.28 | 5.29 | 5.29 |
| 7 | 60.56 | 60.60 | 60.68 |  | 4.95 | 4.92 | 4.88 |
| 8 | 57.56 | 57.64 | 57.75 |  | 4.30 | 4.29 | 4.26 |
| 9 | 61.53 | 61.58 | 61.60 |  | 4.41 | 4.42 | 4.43 |
| 10 | 45.45 | 45.45 | 45.45 |  | 3.61 | 3.61 | 3.60 |
| 11 | 56.41 | 56.44 | 56.49 |  | 4.35 | 4.33 | 4.32 |
| 12 | 62.41 | N.A. | 62.67 |  | 5.05 | N.A. | 5.03 |
| 13 | 60.11 | 60.07 | 60.02 |  | 4.52 | 4.53 | 4.55 |
| 14 | 62.15 | N.A. | 62.16 |  | 4.97 | N.A. | 5.01 |
| 15 | 52.91 | 52.80 | 52.76 |  | 4.78 | 4.79 | 4.80 |
| 16 | 55.03 | 54.90 | 54.83 |  | 4.88 | 4.88 | 4.88 |
| 17 | 58.52 | 58.51 | 58.52 |  | 4.70 | 4.70 | 4.69 |
| 18 | 52.89 | 52.78 | 52.38 |  | 5.06 | 5.09 | 5.14 |
| 19 | 65.40 | 65.47 | 65.53 |  | 4.12 | 4.11 | 4.10 |
| 20 | 57.49 | 57.56 | 57.63 |  | 4.35 | 4.35 | 4.34 |
| 21 | 55.99 | 55.99 | 55.99 |  | 4.68 | 4.67 | 4.66 |
| 22 | 59.72 | 59.74 | 59.82 |  | 4.90 | 4.90 | 4.89 |
| 23 | 62.44 | 62.35 | 62.29 |  | 3.63 | 3.63 | 3.63 |
| 24 | 60.74 | 60.78 | 60.82 |  | 3.88 | 3.87 | 3.84 |
| 25 | 56.03 | 56.10 | 56.17 |  | 4.53 | 4.53 | 4.53 |
| 26 | 67.74 | 67.82 | 67.89 |  | 3.38 | 3.38 | 3.38 |
| 27 | 59.29 | 59.30 | 59.30 |  | 4.59 | 4.61 | 4.63 |
| 28 | 55.45 | 55.52 | 55.57 |  | 4.15 | 4.16 | 4.15 |
| 29 | 59.87 | 59.94 | 59.99 |  | 4.20 | 4.20 | 4.19 |
| 30 | 66.23 | 66.35 | 66.43 |  | 3.49 | 3.49 | 3.48 |
| 31 | 60.12 | 60.16 | 60.18 |  | 3.82 | 3.81 | 3.81 |
| 32 | 57.49 | 57.53 | 57.54 |  | 4.27 | 4.25 | 4.25 |
| 33 | 58.40 | 58.49 | 58.54 |  | 4.30 | 4.30 | 4.30 |
| 34 | 55.43 | 55.41 | 55.38 |  | 4.57 | 4.56 | 4.56 |
| 35 | 46.12 | 46.11 | 46.08 |  | 4.14 | 4.14 | 4.13 |
| 36 | 57.95 | 58.10 | 58.24 |  | 4.41 | 4.41 | 4.41 |
| 37 | 61.59 | 61.63 | 61.63 |  | 4.62 | 4.62 | 4.62 |
| 38 | 66.20 | 66.31 | 66.38 |  | 4.11 | 4.11 | 4.11 |
| 39 | 55.89 | 55.96 | 56.03 |  | 4.41 | 4.40 | 4.38 |
| 40 | 55.66 | 55.64 | 55.60 |  | 4.45 | 4.43 | 4.41 |
| 41 | 56.74 | 56.81 | 56.82 |  | 4.20 | 4.21 | 4.27 |
| 42 | 55.25 | 55.32 | 55.36 |  | 4.48 | 4.47 | 4.46 |
| 43 | 53.10 | 53.09 | 53.13 |  | 5.38 | 5.38 | 5.38 |
| 44 | 59.04 | 58.98 | 58.89 |  | 4.93 | 4.93 | 4.97 |
| 45 | 56.60 | 56.65 | N.A. |  | 5.18 | 5.19 | N.A. |
| 46 | 52.61 | 52.64 | 52.67 |  | 3.70 | 3.71 | 3.71 |
| 47 | 45.45 | 45.48 | 45.50 |  | 3.45 | 3.43 | 3.43 |
| 48 | 54.65 | 54.64 | 54.66 |  | 4.61 | 4.62 | 4.62 |
| 49 | 55.66 | 55.64 | 55.60 |  | 4.45 | 4.43 | 4.41 |
| 50 | 54.29 | 54.22 | 54.15 |  | 4.09 | 4.11 | 4.13 |
| 51 | 56.03 | 56.10 | 55.95 |  | 4.53 | 4.53 | 4.52 |
| 52 | 56.78 | 56.71 | 56.82 |  | 4.37 | 4.29 | 4.27 |
| 53 | 45.46 | 45.47 | 45.45 |  | 4.10 | 4.08 | 4.32 |
| 54 | 54.42 | 54.50 | 54.55 |  | 4.70 | 4.71 | 4.68 |
| 55 | 59.78 | 59.75 | N.A. |  | 5.23 | 5.24 | N.A. |
| 56 | 58.76 | 58.79 | 58.79 |  | 4.05 | 4.07 | 4.07 |
| 57 | 61.17 | 61.20 | 61.24 |  | 4.24 | 4.24 | 4.22 |
| 58 | 57.45 | 57.55 | 57.54 |  | 4.27 | 4.24 | 4.25 |
| 59 | 58.36 | 58.42 | 58.48 |  | 4.64 | 4.65 | 4.66 |
| 60 | 54.26 | 54.27 | 54.30 |  | 4.35 | 4.35 | 4.36 |
| 61 | 62.55 | 62.59 | 62.64 |  | 3.37 | 3.36 | 3.33 |
| 62 | 53.71 | 53.70 | 53.69 |  | 4.48 | 4.47 | 4.45 |
| 63 | 57.96 | 58.00 | 58.00 |  | 3.97 | 3.97 | 3.96 |
| 64 | 58.48 | 58.44 | 58.41 |  | 3.32 | 3.33 | 3.33 |
| 65 | 61.02 | 61.12 | 61.20 |  | 4.64 | 4.63 | 4.62 |
| 66 | 62.57 | 62.63 | N.A. |  | 5.28 | 5.27 | N.A. |
| 67 | 53.88 | 53.87 | N.A. |  | 5.07 | 5.09 | N.A. |
| 68 | 55.18 | 55.12 | 55.10 |  | 5.29 | 5.32 | 5.34 |
| 69 | 53.81 | 53.87 | N.A. |  | 5.18 | 5.20 | N.A. |
| 70 | 60.70 | 60.57 | 60.40 |  | 4.37 | 4.43 | 4.48 |
| 71 | 54.09 | N.A. | N.A. |  | 5.01 | N.A. | N.A. |
| 72 | 55.79 | 55.90 | 56.05 |  | 4.28 | 4.25 | 4.21 |
| 73 | 55.01 | 54.99 | 54.99 |  | 4.38 | 4.38 | 4.36 |
| 74 | 56.54 | 56.71 | 56.82 |  | 4.31 | 4.29 | 4.27 |
| 75 | 45.34 | 45.35 | 45.36 |  | 3.96 | 3.96 | 3.97 |
| 76 | 46.14 | 46.12 | 46.11 |  | 3.80 | 3.81 | 3.81 |

Some values are not available due to overlapping with water signal. Those places are filled with “N.A.”
