# Supplementary material for: Cold-Induced Changes in the Protein Ubiquitin
Source: PLoS One. 2012 Jun 21;7(6):e37270. doi: 10.1371/journal.pone.0037270 (PMC3380907; doi:10.1371/journal.pone.0037270)
Supplement: Table S3 — Carbon and Proton Chemical shifts of ubiquitin methyl groups at 298 K, 278 K and 263 K. (DOC) [file pone.0037270.s008.doc]

**Table S3. Carbon and Proton Chemical shifts of ubiquitin methyl groups at 298K, 278K and 263K.**

| Atom name | Methyl carbon  Chemical shifts(ppm) | | |  | Atom name | Methyl proton  Chemical shifts(ppm) | | |
| --- | --- | --- | --- | --- | --- | --- | --- | --- |
|  | 298K | 278K | 263K |  |  | 298K | 278K | 263K |
| I3Cδ1 | 14.31 | 14.40 | 14.47 |  | I3Hδ1 | 0.58 | 0.59 | 0.58 |
| I3Cγ2 | 17.94 | 17.90 | 17.87 |  | I3Hγ2 | 0.62 | 0.62 | 0.61 |
| V5Cγ1 | 22.44 | 22.42 | 22.31 |  | V5Hγ1 | 0.68 | 0.67 | 0.66 |
| V5Cγ2 | 20.91 | 20.92 | 20.95 |  | V5Hγ2 | 0.72 | 0.72 | 0.71 |
| T7Cγ2 | 21.56 | 21.56 | 21.50 |  | T7Hγ2 | 1.17 | 1.17 | 1.16 |
| L8Cδ1 | 25.47 | 25.55 | 25.64 |  | L8Hδ1 | 1.04 | 1.04 | 1.02 |
| L8Cδ2 | 23.89 | 23.98 | 24.02 |  | L8Hδ2 | 0.97 | 0.98 | 0.97 |
| T9Cγ2 | 21.98 | 21.99 | 22.00 |  | T9Hγ2 | 1.27 | 1.26 | 1.25 |
| T12Cγ2 | 22.06 | 22.14 | 22.20 |  | T12Hγ2 | 1.07 | 1.07 | 1.06 |
| I13Cδ1 | 14.52 | 14.63 | 14.70 |  | I13Hδ1 | 0.71 | 0.72 | 0.72 |
| I13Cγ2 | 17.84 | 17.87 | 17.91 |  | I13Hγ2 | 0.87 | 0.88 | 0.87 |
| T14Cγ2 | 21.87 | 21.87 | 21.89 |  | T14Hγ2 | 1.13 | 1.13 | 1.13 |
| L15Cδ1 | 27.17 | 27.36 | 27.50 |  | L15Hδ1 | 0.70 | 0.70 | 0.69 |
| L15Cδ2 | 24.23 | 24.11 | 24.02 |  | L15Hδ2 | 0.76 | 0.76 | 0.75 |
| V17Cγ1 | 22.27 | 22.20 | 22.18 |  | V17Hγ1 | 0.70 | 0.69 | 0.68 |
| V17Cγ2 | 19.65 | 19.80 | 19.92 |  | V17Hγ2 | 0.42 | 0.40 | 0.38 |
| T22Cγ2 | 22.35 | 22.34 | 22.32 |  | T22Hγ2 | 1.25 | 1.26 | 1.25 |
| I23Cδ1 | 9.41 | 9.31 | 9.23 |  | I23Hδ1 | 0.78 | 0.78 | 0.77 |
| I23Cγ2 | 18.19 | 18.14 | 18.08 |  | I23Hγ2 | 0.69 | 0.69 | 0.67 |
| V26Cγ1 | 21.57 | 21.62 | 21.62 |  | V26Hγ1 | 0.97 | 0.98 | 0.97 |
| V26Cγ2 | 23.66 | 23.73 | 23.80 |  | V26Hγ2 | 1.62 | 1.63 | 1.62 |
| I30Cδ1 | 15.27 | 15.54 | 17.84 |  | I30Hδ1 | 0.88 | 0.90 | 0.90 |
| I30Cγ2 | 17.15 | 17.19 | 17.24 |  | I30Hγ2 | 0.69 | 0.70 | 0.70 |
| I36Cδ1 | 13.69 | 13.89 | 14.05 |  | I36Hδ1 | 0.79 | 0.80 | 0.80 |
| I36Cγ2 | 17.78 | 17.73 | 17.67 |  | I36Hγ2 | 0.93 | 0.94 | 0.94 |
| L43Cδ1 | 26.50 | 26.65 | 26.73 |  | L43-Hδ1 | 0.76 | 0.76 | 0.75 |
| L43Cδ2 | 24.30 | 24.40 | 24.54 |  | L43Hδ2 | 0.79 | 0.79 | 0.78 |
| I44Cδ1 | 12.83 | 12.80 | 12.68 |  | I44Hδ1 | 0.67 | 0.67 | 0.66 |
| I44Cγ2 | 17.66 | 17.71 | 17.71 |  | I44Hγ2 | 0.68 | 0.68 | 0.67 |
| L50Cδ1 | 26.04 | 26.16 | 26.27 |  | L50Hδ1 | 0.51 | 0.52 | 0.52 |
| L50Cδ2 | 19.65 | 19.55 | 19.52 |  | L50Hδ2 | -0.17 | -0.19 | -0.20 |
| T55Cγ2 | 22.37 | 22.45 | 22.50 |  | T55Hγ2 | 1.12 | 1.11 | 1.10 |
| L56Cδ1 | 26.87 | 26.97 | 27.04 |  | L56Hδ1 | 0.74 | 0.74 | 0.73 |
| L56Cδ2 | 23.20 | 23.21 | 23.24 |  | L56Hδ2 | 0.61 | 0.62 | 0.61 |
| I61Cδ1 | 14.52 | 14.90 | 15.20 |  | I61Hδ1 | 0.41 | 0.44 | 0.45 |
| I61Cγ1 | 28.29 | 28.40 | 28.27 |  | I61Hγ12 | -0.39 | -0.49 | -0.50 |
| I61Cγ2 | 17.36 | 17.46 | 17.56 |  | I61Hγ2 | 0.47 | 0.47 | 0.46 |
| T66Cγ2 | 21.54 | 21.62 | 21.68 |  | T66Hγ2 | 0.93 | 0.92 | 0.91 |
| L67Cδ1 | 24.95 | 24.99 | 25.01 |  | L67Hδ1 | 0.69 | 0.69 | 0.68 |
| L67Cδ2 | 25.32 | 25.32 | 25.28 |  | L67Hδ2 | 0.65 | 0.66 | 0.65 |
| L69Cδ1 | 24.02 | 24.03 | 24.08 |  | L69Hδ1 | 0.86 | 0.87 | 0.88 |
| L69Cδ2 | 26.20 | 26.18 | 26.06 |  | L69Hδ2 | 0.74 | 0.76 | 0.78 |
| V70Cγ1 | 21.54 | 21.62 | 21.68 |  | V70Hγ1 | 0.93 | 0.92 | 0.91 |
| V70Cγ2 | 20.75 | 20.59 | 20.36 |  | V70Hγ2 | 0.83 | 0.82 | 0.80 |
| L71Cδ1 | 25.14 | 25.16 | 25.15 |  | L71Hδ1 | 0.96 | 0.97 | 0.97 |
| L71Cδ2 | 24.02 | 24.03 | 24.35 |  | L71Hδ2 | 0.86 | 0.87 | 0.86 |
| L73Cδ1 | 25.09 | 25.16 | 25.22 |  | L73Hδ1 | 0.92 | 0.93 | 0.93 |
| L73Cδ2 | 23.44 | 23.39 | 23.34 |  | L73Hδ2 | 0.87 | 0.87 | 0.86 |
